# Foramen Changes following Over Instrumentation of Curved Canals with Three Engine-Driven Instruments: An *In Vitro *Study

**DOI:** 10.22037/iej.v12i4.16268

**Published:** 2017

**Authors:** Salwa Yammine, Edgard Jabbour, Paul Nahas, Zeina Majzoub

**Affiliations:** a *Department of Endodontics, Lebanese University, Campus Rafic Hariri, Hadath, Beirut, Lebanon; *; b *Department of Endodontics, Lebanese University, Campus Rafic Hariri, Hadath, Beirut, Lebanon; *; c *Department of Restorative and Esthetic Dentistry, Lebanese University, Campus Rafic Hariri, Hadath, Beirut, Lebanon; *; d * Department of Periodontology, Lebanese University, Campus Rafic Hariri, Hadath, Beirut, Lebanon*

**Keywords:** Apical Foramen, Curved Canals, Foramen Area, Foramen Shape, Nickel Titanium Rotary Instruments, Over Instrumentation

## Abstract

**Introduction:**

The present *in vitro* study aimed to evaluate and compare the changes in shape and surface area of the major foramen following shaping of curved canals with three new generation NiTi engine-driven instruments naming ProTaper Next, BT RaCe and WaveOne Gold- with 3 different levels of protrusion beyond the major apical foramen.

**Methods and Materials:**

A total of 45 extracted human molars with at least one curved canal were distributed in 3 comparable groups of 15 that were instrumented using either ProTaper Next (PTN), BT RaCe (BTR) or WaveOne Gold (WOG). The canals were instrumented to the major foramen and then over instrumented with the final file 0.5 mm, 1 mm and 1.5 mm beyond the foramen. Standardized pre- and post-instrumentation photographs of the foramen were obtained for all groups using a stereomicroscope. Foramen shape and surface area were evaluated using the AmScope software for measurements and compared between groups and levels of instrumentation applying binary conditional logistic regression and repeated measures ANOVA. The level of significance was set at 0.05.

**Results:**

Foramen shape tended to gradually change from circular to oval as the level of instrumentation increased in all groups. The original foramen shape in WOG group remained better than other groups. Foramen surface areas increased in all groups with Group BTR demonstrating significantly greater values than the other 2 groups.

**Conclusion:**

Over instrumentation resulted in apical enlargement and ovalization in all 3 groups but with different patterns. These differences can be attributed to the final file size, design characteristics and kinematics of the 3 systems.

## Introduction

Available literature has demonstrated that the variability and anatomical complexity of the apical region make it difficult to adequately shape and clean the root canal system maintaining the original position, shape and diameter of the major apical foramen. The apical constriction is the ideal termination point of canal shaping and obturation as the natural narrowing of the root canal and practically the termination of the pulpal tissue [1]. By applying the apical constriction as the terminus for root canal therapy, the apical foramen is not altered by instrumentation as it is only negotiated by the patency file. Since the apical constriction may not always be present or detectable [2-4], clinicians tend to use the position of the major apical foramen as the landmark for instrumentation and obturation [1, 5]. The drawbacks of using the apical foramen as the reference apical limit are related to difficulties in identifying the true anatomical apex which is 0 to 3.0 mm short from the radiographic apex [6].

**Figure 1:**
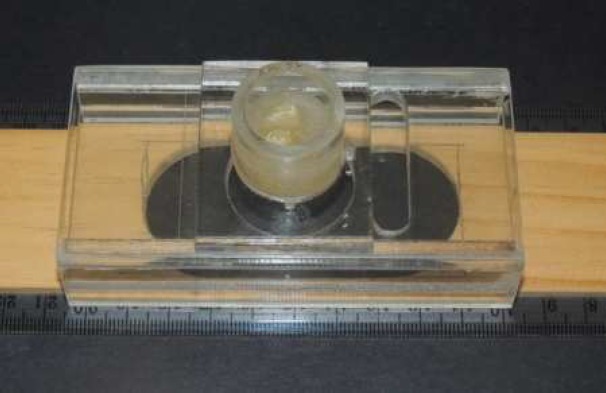
Plexiglas cylinder containing a centrally positioned specimen with the apices facing upward. The cylinder is fixed onto a prefabricated Plexiglas mount to standardize radiographic and photographic documentation. The T-ruler serves to slide the mounting

In addition to these problems in identifying the apical anatomical landmarks, controversies exist regarding the selection of the ideal termination of root canal therapy in teeth with vital or infected pulps. While some authors recommend terminating the endodontic procedures short of the apical foramen in cases of vital or infected pulp [7], recent investigations tend to extend the length of root canal instrumentation to or beyond the foramen for efficient disinfection in cases of apical periodontitis [8].

Whether over instrumentation is intentional or unintentional, it results in enlargement and shape modification of the major apical foramen [9, 10]. As a result, various irrigants and filling materials, bacteria and their byproducts and dentinal debris are likely to extrude into the periradicular tissues with subsequent postoperative pain [11], non hermetic seal of the foramen, delayed apical healing, formation of apical cracks and zipping [12].

Nickel-titanium (NiTi) engine-driven systems with improved cutting efficiency, rotational resistance and flexibility have been reported to result in less straightening and better centered preparations of curved root canals [13, 14]. The newer fifth generation of NiTi rotary systems, include ProTaper Next (PTN) instruments (Dentsply Maillefer, Ballaigues, Switzerland), WaveOne Gold®(WOG) (Dentsply Maillefer, Ballaigues, Switzerland), and BT-Race®(BTR) (FKG Dentaire, La Chaux-de-Fonds, Switzerland).

PTN instruments are made from M-wire and are characterized by multiple progressive and regressive percentage tapers and an innovative off-centered rectangular cross section that allows a swaggering movement as they advance into the root canal [15]. The different percentage tapers result in a decreased screw effect and reduce the incidence of taper lock by minimizing the contact between the file and dentin [16]. 

WOG reciprocating file was developed as a single file technique with post-manufacturing gold process in which the ground NiTi file is heat-treated and slowly cooled. The cross-section is a parallelogram with 85-degree cutting edges alternating with an off-centered cross-section where only two cutting edges touch the canal wall. The kinematics of the WOG systems is represented by a reciprocating movement with 170^°^ counterclockwise and 50^°^ clockwise rotation with 120^°^ of difference between the 2 movements. This NiTi system has improved flexibility, resistance to cyclic fatigue and cutting efficiency [17].

The BTR system is designed to treat the majority of canals with a sequence of 3 files including BT1 (10/0.06), BT2 (35/0.00), and BT3 (35/0.04). As reported by the manufacturer, BTR files are provided with a “Booster Tip” (BT) which increases the files efficiency [18].

Currently, there is limited information regarding the effects of over instrumentation with NiTi rotary systems on foramen morphological alterations [14, 15, 19] and none with the fifth generation of instruments.

The objectives of the present *in vitro* study are to compare the morphological changes in shape and surface area of the major foramen following over instrumentation with three aforementioned instruments- ProTaper Next, BT RaCe and WaveOne Gold- in curved canals with the instruments protruding at different levels (0.5 mm, 1 mm and 1.5 mm) beyond the apex. The null hypothesis is that there is no statistical difference in foramen changes following over instrumentation with these instruments used in canals with moderate to severe curvature.

## Materials and Methods

The study was approved by the Ethics Committee of the Lebanese University, Hadath, Lebanon (No. 137/14).The study protocol and informed consent were in full accordance with the ethical principles of the Declaration of Helsinki of 1975 as revisited in 2000. Forty-five extracted human maxillary and mandibular molars were selected for the study based on the following inclusion criteria: 1) complete apex formation; 2) absence of apical resorption or cracks; 3) presence of at least one canal with a moderate to severe angle of curvature (20^°^ to 40^°^) as measured radiographically according to Schneider’s technique [20]; 4) no history of endodontic treatment; and 5) foramen surface area ranging between 15 mm^2^ and 25 mm^2^ as measured on digital photographs. The patients were informed about the study objectives and procedures and their informed written consent to use their extracted teeth for research purposes was obtained. 

Immediately after extraction, the teeth were cleaned under a continuous water flow and all debris from the periodontal ligament were eliminated using an ultrasonic device. Subsequently, the teeth were stored at room temperature in a 0.9% normal saline solution supplemented with 0.1 % thymol (Sigma Chemical Co., St Louis, MO, USA) for antibacterial activity [9, 21]. The storage solution was changed weekly to maintain cleanliness, hydration and disinfection.

All crowns were sectioned coronally above the cemento-enamel junction in order to obtain roots of uniform 16 mm length. Standardized access cavities were made and sealed with cotton pellets and modeling wax. Subsequently, the specimens were positioned in the center of a 2 cm high Plexiglas cylinder with the apices facing upward (Figure 1). Fast setting acrylic resin (Paladur Heraeus Kulzer, Inc., South Bend, IN, USA) was poured into the cylinder leaving 2 mm of the apices uncovered. After setting, the cylinders were fixed onto a customized Plexiglas device mounted on a wooden T-ruler (Figure 1). This device was used to standardize the photographic documentation of the apical foramen throughout the experimental procedures. Furthermore, the positional changes of the T-ruler served to slide the mounting device relative to the imaging microscope and place the apices into focus. A baseline radiograph was taken to confirm the absence of cracks and the presence of canals with moderate to severe curvature.

***Canal preparation***

The cylinders containing the specimens were transferred into a second prefabricated light cured resin mount to stabilize the samples during canal preparation. One mesiobuccal or mesiolingual curved canal of the mesial root was treated while the remaining canals were left uninstrumented. The canals were localized using an endodontic probe (DG16, Hu Friedy, USA) and negotiated with a #8 or 10 K-file (FlexoFile, Dentsply Maillefer, Ballaigues, Switzerland) in the presence of a viscous chelator Glyde (Glyde File Prep, Dentsply Maillefer, Ballaigues, Switzerland) until the tip was just visible and tangent to the apical foramen under a stereomicroscope (SM-1TSZZ-144S, Irvine, CA, USA) under 45× magnification. The silicone stop was adjusted to the nearest flat anatomical landmark and the distance between the file’s tip and the rubber stopper was measured with an endodontic ruler (Dentsply Maillefer, Ballaigues, Switzerland). The working length (WL) was recorded as the reference landmark file tip measurement minus 0.5 mm. 

***Experimental design and canal preparation***

The 45 selected canals were randomly assigned to 3 groups according to the type of engine-driven NiTi system: group PTN (*n*=15), group BTR (*n*=15) and group WOG (*n*=15). A manual glide path was created with #10 and 15 K-File for the BTR group while a mechanical glide path was obtained with rotary ProGlider 0.16 file (PG, Dentsply Maillefer, Ballaigues, Switzerland) for the other two groups. The preprogrammed endo motor (X-Smart Plus, Dentsply, Maillefer, Ballaigues, Switzerland) was set for each group of NiTi instruments using the related specific settings (torque, speed and movement) recommended by the manufacturer. One set of new instruments was used for each canal. During instrumentation, canals were irrigated using 3 mL of 5% NaOCl solution after each file and the Glyde chelator was used as lubricant. Final irrigation was carried out with 1 mL of 17% EDTA for 1 min, then by a final rinse of 3 mL of NaOCl. The final apical preparation was standardized to size 25 for the PTN and WOG instruments and to size 35 for BTR. Root canal instrumentation was performed by one single experienced endodontist.

In all specimens, the canals were instrumented to the working length, then to the major foramen (WL plus 0.5 mm), and subsequently over instrumented up to 0.5 mm, 1 mm and 1.5 mm beyond the apex. The working length was recalculated when increasing the file sizes and levels of instrumentation/over instrumentation to avoid file protrusion beyond the desired amount.

Table 1 summarizes the instrumentation and over instrumentation sequences in the 3 groups.

***Image*** ***analysis*** ***and measurements***

The major foramen of all specimens were photographed before (baseline) and after different levels of instrumentation (to the foramen, 0.5, 1 and 1.5 mm beyond the apex). Photographs were take under 45× magnification using a digital camera (14MP USB digital camera, MU1403) coupled to the stereomicroscope. Photographic documentation was performed under standardized conditions using the Plexiglas mounting device where the cylinders were transferred after each level of instrumentation. The apices were illuminated directly and oriented using the T-ruler so that the foramen was located in the middle of and parallel to the objective lens.

The digital images were transferred to a personal computer and the foramen surface area was evaluated on each photograph using the AmScope image analysis software (AmScope, Irvine, CA, USA). The surface was measured after outlining manually the foramen boundary and expressed in mm^2^ with 3 decimal digits. The general shape of the foramen was assessed as round when the difference between the wide and narrow diameters was less than 0.02 mm or oval for values equal or exceeding 0.02 mm [22]. 

**Figure 2:**
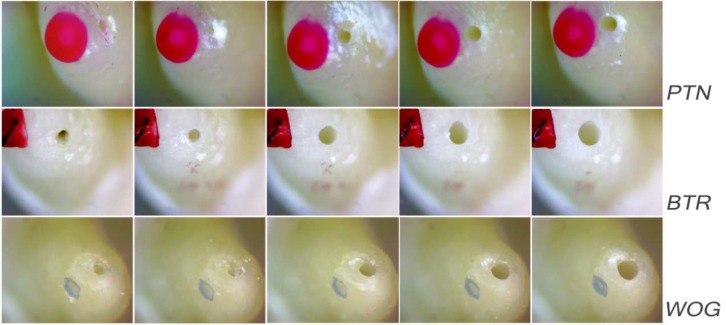
Example of foramen in each of the 3 groups demonstrating shape and surface area modifications from left to right: baseline, foramen, 0.5 mm, 1 mm, and 1.5 mm levels of over instrumentation

Post-instrumentation photographs were superimposed onto baseline images to evaluate changes in foramen area. Precision of superimposition was achieved by marking the apical surface close to the foramen with a small spot of utility wax (Figure 2). Intra-observer reliability was calculated based on 10 repeated area measurements of 5 canals prior to and following instrumentation. High intra-observer agreement with intra-class correlation coefficient of 0.96 was calculated. 

Statistical analysis was performed using SPSS Statistical Package for Social Sciences version 21.0 (SPSS, Inc., Chicago, IL, USA). Descriptive statistics with means, standard deviations and frequency distributions were reported. Binary conditional logistic regression was used to check the effect of group and level on the changes of foramen shape. Repeated measures (ANOVA) were used to compare the foramen surface area between groups at the various levels (pre-instrumentation baseline, instrumentation at the foramen, at 0.5 mm, at 1 mm and at 1.5 mm). *P*-value was set at 0.05 for statistical significance.

## Results

Summary of foramen shape changes are presented in table 2. 

The 2 main significant findings confirmed using binary conditional logistic regression approach with groups as dummy variables (*P*<0.0001 between levels; *P*<0.029 for groups) are: first the tendency for foramen shape to gradually change from circular to oval as the level of instrumentation increased and second the different trend in shape modification between groups. Changes in foramen shape in group PTN started when instrumentation was at the foramen level but the foramen fairly maintained the same shape as the level of over instrumentation increased. In the BTR group, changes were evident at the 0.5 mm level but substantially increased at the 1 mm level. The canals in group WOG maintained the original circular shape at almost all levels of over instrumentation. 

**Table 1:** Sequence of instruments used for canal preparation in the 3 groups PTN, BTR and WOG

	**PTN Group**	**BTR Group**	**WOG Group**
**Canal Negotiation**	8-10 K-file	8-10 K-file	8-10 K-file
**Instrument to Working Length**	ProGlider	10 and 15 K-file 10.06 BT1	ProGlider
**Instrument to Foramen**	X1 X2	35.00 BT2 35.04 BT3	Primary
**0.5 mm beyond Foramen**	X2	35.04 BT3	Primary
**1 mm beyond Foramen**	X2	35.04 BT3	Primary
**1.5 mm beyond Foramen**	X2	35.04 BT3	Primary

**Table 2:** Frequency distribution of canals according to shape following different levels of instrumentation in the 3 groups

	**PTN Group**		**BTR Group**		**WOG Group**	
**Non Instrumented Canal**	8 (53.3%)	7 (46.7%)	9 (60.0%)	6 (40.0%)	9 (60.0%)	6 (40.0%)
**Instrument to Foramen**	5 (33.3%)	10 (66.7%)	8(53.3%)	7 (46.7%)	9 (60.0%)	6 (40.0%)
**0.5 mm beyond Foramen**	4 (26.7%)	11 (73.3%)	5(33.3 %)	10 (66.7%)	8 (53.3%)	7 (46.7%)
**1 mm beyond Foramen**	4 (26.7%)	11 (73.3%)	1(6.7 %)	14 (93.3%)	4 (26.7%)	11 (73.3%)
**1.5 mm beyond Foramen**	3 (20.0%)	12 (80.0%)	2 (13.3%)	13 (86.7%)	7 (46.7%)	8 (53.3%)

**Table 3:** Surface area of the foramen in mm^2^ according to group and level of instrumentation

	**PTN Group**	**BTR Group**	**WOG Group**	***P*** **-** **value**
**Non Instrumented Canal**	0.022±0.011	0.024±0.010	0.018±0.006	0.237a
**Instrument to Foramen**	0.027± 0.012	0.035±0.014	0.024±0.007	
**0.5 mm beyond Foramen**	0.036±0.012	0.060±0.020	0.042±0.013	
**1 mm beyond Foramen**	0.046±0.015	0.085±0.025	0.058±0.016	
**1.5 mm beyond Foramen**	0.056±0.016	0.108±0.024	0.075±0.022	
***P*** **-value**	<0.0001^b^	<0.0001^c^	<0.0001^d^	

Table 3 summarizes foramen surface areas according to over instrumentation levels and groups. Based on repeated measures ANOVA the three groups were significantly different (*P*<0.0001). The measures between the five levels were significantly different (*P*<0.0001) and group levels were significantly different (*P*<0.0001). However, the foramen surface area were not significantly different between groups at baseline (non-instrumented canals) using one way ANOVA (*P*=0.237).

## Discussion

The aim of the present study was to assess and compare foramen changes following over instrumentation with 3 different rotary NiTi systems ProTaper Next®, BT RaCe® and WaveOne Gold® in curved canals of extracted human molar teeth. The null hypothesis was rejected in that significant differences were demonstrated between the 3 systems relative to both shape and surface variables.

The extracted human teeth model used in the present study along with operator-driven instruments (and not computer-driven) provide conditions that simulate clinical situations with adjustment of digital pressure according to canal anatomy and perceived stress on the endodontic files [23]. Although the mounts are not identical to setups used in other studies [10, 19, 24], they similarly allow standardization of image capturing thus providing the means for accurate comparisons of repeated measurements. Care was taken during sample selection to include teeth with comparable foramen size as confirmed by the absence of statistically significant differences in foramen surface at baseline between the 3 groups. The selection of canals with moderate to severe curvature was based on the assumption that curved canals are more likely to display substantial changes in foramen morphology following instrumentation and over instrumentation.

Unintentional over instrumentation is commonly observed during endodontic procedures even when electronic apex locators are used [7]. Although these devices are significantly more accurate in identifying the WL [25], they are still often associated with over instrumentation and extension of endodontic files beyond the major foramen [26, 27]. In addition, the use of rotary NiTi systems is coupled with lack of absolute control of the working length during root canal instrumentation with subsequent protrusion of files beyond the foramen [10, 28, 29]. Furthermore, per-operative changes in the working length are likely to occur in curved roots due to canal straightening [29, 30]. These findings are in agreement with those of the present investigation where a change in working length of 0.4-0.5 mm was observed during cleaning and shaping. 

In the present study, the original shape of the major foramen was circular in more than half of the specimens (26 out of 45). This finding is in accordance with the results of Gani and Virvician and Martos *et al. *[31, 32] but in disagreement with other investigators who reported that the oval shape was most commonly found [22, 33-35]. These discrepancies can be attributed to multiple factors such as differences in age of the donor, sample size, method used to assess shape and other biomechanical factors [35]. Following instrumentation to the foramen, a drastic change from circular to oval shape was observed in the PTN group with approximately 38% (3 out of 8) of the originally circular foramen becoming oval. This trend continued at the following levels of over instrumentation with 50% (4 out of 8) of the initially round foramen becoming oval at the 1.5mm level. This shape alteration is likely to be related to the offset design of the system and its kinematics with the characterizing snake-like swaggering movement [36]. In the BTR group, the trend was similar but with nearly 11% (1 out of 9) of the canals changing from the original circular to the oval shape when instrumentation reached the foramen. A drastic reduction in the proportion of round canals was observed at 0.5 mm protrusion and continued with the successive levels of over instrumentation. At the 1.5 mm level, nearly 78% (7 out of 9) of the initially circular foramen became oval in the BTR group. This finding could be attributed to the file design which alternates effective cutting parts with an open pitch cylindrical part that allows the instrument to collect debris. It is likely that ovalization of the foramen starts when the cylindrical part of the flute engages the foramen with a tendency to touch the foraminal walls in a non-uniform pattern circumferentially. In the WOG group, nearly 78% (7 out of 9) of the originally circular foramen maintained their round shape up to the final level of over instrumentation. It is difficult to attribute this finding to a single kinematic factor, rotational speed, or design-related characteristics since the reciprocating type of NiTi files have not been reported to yield better centralization than the continuous instruments in curved canals [37]. Analysis of such factors is beyond the scope of the present investigation.

Foramen ovalization has been reported following canal over instrumentation with a variety of NiTi rotary instruments [38]. Although no studies have evaluated the specific impact of foramen ovalization on the quality of the apical seal, it can be tentatively speculated that better apical sealing can be achieved with round foramen since they better match the shape of the round gutta-percha master cone [39]. In order to ensure adequate cleaning and sealing of the endodontic system, the morphology of the foramen should be modified towards the round shape, or the instruments and techniques should be adapted to non-round contours [35].

Although consistent negative outcomes of over instrumentation have not been documented clinically, protrusion of rotary NiTi files beyond the major foramen is likely to be associated with apical enlargement [40], more postoperative pain [13], destruction of the apical stop [41], and extrusion of necrotic debris and microorganisms with their byproducts with potential persistent infection [7]. Conversely, Silva* et al. *[35] concluded that although apical enlargement following 1 mm over instrumentation beyond the foramen resulted in more apical deviation, it however yielded better cementum removal and better apical seal. Furthermore, bacterial microleakage was not significantly different between enlarged and non-enlarged foramen [35]. In cases of apical periodontitis, larger apical preparations have been advocated to optimize root canal disinfection [42], and result in better healing of chronic periapical lesions [43]. In the present study, increasing foraminal surface area was observed with increasing levels of over instrumentation in all 3 groups of NiTi systems. To our knowledge, only one previously published study assessed changes in foramen area with NiTi files protruding 1 mm beyond the WL [38]. 

The results of the present investigation demonstrated an increase in mean foramen surface from 0.022 mm² at baseline to 0.056 mm² at the final level of over instrumentation for the PTN group, from 0.024 mm² to 0.108 mm² for the BTR group and from 0.018 mm² to 0.075 mm² for the WOG group. Foramen surface was not significantly different between the three groups at baseline. After instrumentation and over instrumentation the three groups were significantly different in terms of foraminal surface area. This finding is related to the fact that the final file diameter of the BTR instruments at the working length and all following levels is larger than that of the other 2 groups. It is important to emphasize that the final instrument selection in all 3 groups was based on their respective manufacturers’ recommendations. In addition to the larger final file diameter, other design and kinematic variables could account for the significantly more enlarged foramen observed in the BTR group. The tendency, which was found to be statistically significant, towards less apical enlargement with the PTN system might be related to the rectangular cross section of the files consenting contact with the dentinal walls at two points at a time. While the snake-like movement of the PTN files might have a negative impact on the ovalization of major foramen, the cross-section of these files is likely to result in less apical widening. Further studies are required to assess the impact of over instrumentation on apical widening with similar size files to focus on files design and kinematics as the sole confounding variable.

## Conclusion

According to the results of the present study, the 3 types of NiTi systems ProTaper Next, BT RaCe and WaveOne Gold resulted in shape and surface area modifications following over instrumentation in curved canals. The ProTaper Next and WaveOne Gold resulted in less apical widening than the BT RaCe instruments while the WaveOne Gold maintained better the original shape of the foramen than the other two systems. Minor alterations in foramen surface area were demonstrated with the 3 systems when instrumentation reached the major foramen, however more drastic changes were evident when instruments protruded 0.5 mm and more beyond the foramen. These changes should be taken into account whether apical enlargement is a desirable end-point of endodontic therapy or not.
